# *Staphylococcus aureus* transcriptomics and single-cell sequencing approaches

**DOI:** 10.1128/iai.00411-25

**Published:** 2025-09-15

**Authors:** Natalia Malachowa, Frank R. DeLeo

**Affiliations:** 1 Laboratory of Bacteriology, Rocky Mountain Laboratories, Division of Intramural Research, National Institute of Allergy and Infectious Diseases, National Institutes of Health 2511, Hamilton, Montana, USA; University of California Davis, Davis, California, USA

**Keywords:** RNA-seq, single-cell sequencing, microarrays, transcriptomics

## Abstract

*Staphylococcus aureus* is an important cause of human infections globally and ranks among the top causes of death by bacteria. In addition, the microbe is notorious for developing resistance to antibiotics. Methicillin-resistant *S. aureus* is endemic in healthcare facilities and the community in many regions of the world. Although our understanding of *S. aureus* as a human commensal organism and opportunistic pathogen remains incomplete, the use of genomics and transcriptomics approaches for *S. aureus* research has advanced this knowledge significantly over the past 20 years. This article reviews genomics approaches, with special emphasis on transcriptomics and single-cell sequencing, used to study *S. aureus*, past and present, and highlights selected discoveries made with these methods and new applications moving forward.

## INTRODUCTION

*Staphylococcus aureus* is a gram-positive facultative anaerobic bacterium that typically resides on skin and in the upper respiratory tract as part of normal human microflora (1, 2). At the same time, it is an opportunistic pathogen and one of the leading causes of bacterial infections worldwide (3, 4). *S. aureus* is an etiological agent of many diseases and syndromes, ranging from skin and soft tissue infections to life-threatening endocarditis, osteomyelitis, and respiratory and bloodstream infections (3). The microbe produces numerous virulence factors that facilitate host immune evasion and promote disease (5), yet it is antibiotic resistance that hinders treatment of infections. *S. aureus* develops resistance to antibiotics readily, and resistance tracks closely with the introduction and use of antibiotics. For example, *S. aureus* isolates carrying β-lactamase resistance encoded by *blaZ* (penicillin-resistant *S. aureus*) were detected in the early 1940s, shortly after penicillin was introduced for treatment of infections (6). Methicillin, a semisynthetic β-lactam antibiotic, was brought into clinical use in the late 1950s, and methicillin-resistant *S. aureus* (MRSA) was detected soon afterward (7, 8). MRSA spread worldwide (became pandemic) and is now endemic in both healthcare and community settings. Some MRSA strains are resistant to several additional classes of antibiotics, and treatment options are limited (9). Vancomycin remains an important antibiotic for the treatment of severe *S. aureus* infections, and few vancomycin-resistant *S. aureus* (VRSA*, vanA*-mediated resistance) have been reported to date (10). The relative paucity of VRSA is likely due to the reported fitness cost of this type of resistance and/or instability of the mobile genetic element that encodes resistance in *S. aureus* (11, 12). However, the use of vancomycin or beta-lactam antibiotics can select for *S. aureus* with reduced sensitivity to vancomycin, and these strains have been termed vancomycin-intermediate *S. aureus* (VISA) (13, 14). VISA typically develops from vancomycin-susceptible *S. aureus* via heterogeneous VISA (hVISA) as a result of point mutations that accumulate in the presence of antibiotics (15, 16). hVISA is a mixed population of cells with different levels of susceptibility to vancomycin. hVISA and VISA are typically associated with persistent infection, hospitalization, and/or failure of vancomycin therapy (17, 18).

In 2019, *S. aureus* ranked fifth among all pathogens globally (after tuberculosis, malaria, HIV/AIDS, and *Streptococcus pneumoniae*) for disability-adjusted life-years (34.5 million) and was associated with 1.1 million deaths (3, 19). According to the Centers for Disease Control and Prevention, the number of invasive MRSA infections in the United States decreased by ~21% between 2013 and 2017 (20). Nonetheless, invasive MRSA infections in 2017 accounted for 323,700 cases in hospitalized patients and resulted in an estimated $1.7 billion in healthcare costs (20). The significant decline in U.S. MRSA infections since 2005, especially among hospital-acquired infections (HAIs) (21, 22), may have been attributed to improved strategies to prevent MRSA transmission (23), standardized treatment guidelines (24), as well as a decrease in infections caused by USA100 (CC5), which was the most prominent healthcare-associated MRSA clone (9). Although MRSA remains a common cause of HAI globally, recent reports indicate that methicillin-susceptible *S. aureus* (MSSA) causes more hospital and community bloodstream infections in many geographic regions (22, 25–30).

Molecular epidemiology (molecular typing) delineates the geographical spread and evolution of *S. aureus*, which is fundamental for infection control and prevention. Phage typing (31, 32) was one of the first systematic methods used to track *S. aureus* strains and outbreaks (33, 34). This typing method was used for many decades (1940s–1990s) until the introduction of multilocus enzyme electrophoresis (MLEE) and pulsed-field gel electrophoresis (PFGE). MLEE indexes genetic variation that accumulates slowly (macroevolution) and was therefore used to study the evolution of *S. aureus* (35). By comparison, PFGE tracks recent genetic changes (microevolution) and is used to investigate outbreaks/epidemics (36, 37). McDougal and colleagues established a U.S. national database of *SmaI* PFGE patterns and associated pulsed-field types for *S. aureus* in 2003, thereby standardizing PFGE analyses of *S. aureus* (36). PFGE has high discriminatory power and has been used to study *S. aureus* outbreaks in the U.S. for more than two decades. A PCR-based method known as multiple-locus variable-number tandem repeat analysis (MLVA) has application similar to that of PFGE for tracking *S. aureus* outbreaks and, in comparison, is inexpensive and less technically demanding (38, 39). Although MLVA is used less frequently than PFGE, the method is still used in selected regions of the world for tracking *S. aureus* outbreaks (40). Two additional widely used classification techniques, multilocus sequence typing (MLST) (41, 42) and *spa* typing (43, 44), provide unbiased data analysis and are compatible with creating standardized reference data repositories. MLST assigns an allelic profile or sequence type based on sequence analysis of ~450 bp fragments of seven housekeeping genes (42). MLST indexes variation that accumulates over a long period of time and is useful for studying the evolution of *S. aureus*. Spa typing, which is based on the DNA sequence of the variable number tandem repeat region in the gene encoding protein A (*spa*), is unique in that it provides data that bear on both recent and long-term evolution of *S. aureus* (44). Collectively, these typing methods have been essential for tracking *S. aureus* outbreaks, infection control, and gaining an enhanced understanding of *S. aureus* evolution and virulence. PFGE, MLST, MLVA, and *spa* typing remain in use for *S. aureus* research, but they are being updated or replaced rapidly by whole-genome sequencing (WGS) (45–50).

## WHOLE-GENOME SEQUENCING

**Fig 1:**
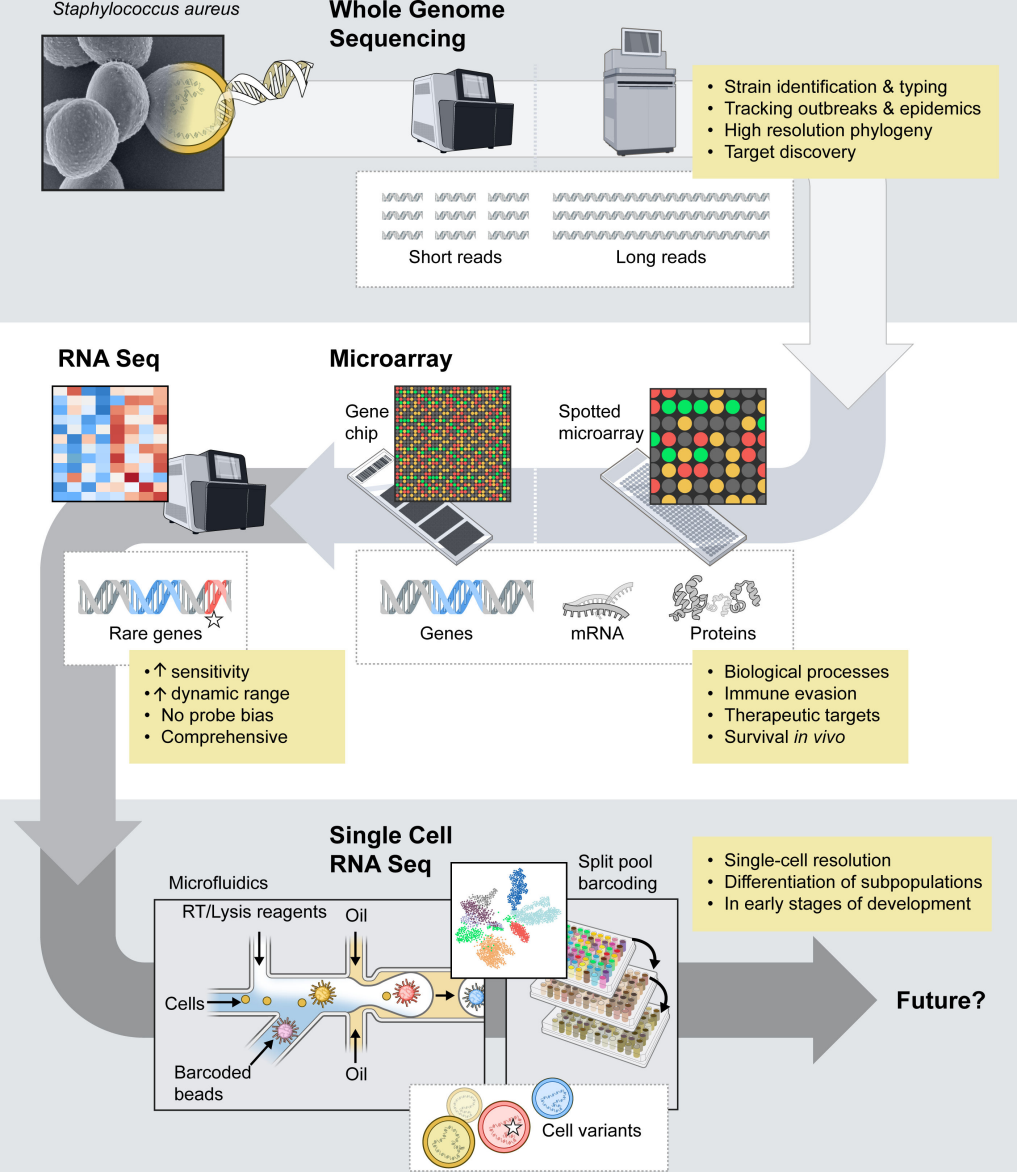
History of *S. aureus* genomics and selected discoveries.

WGS provides comprehensive information about the genetic makeup of microorganisms. In addition to generating complete DNA sequences of microbial genomes, WGS plays a crucial role in clinical microbiology and public health by contributing to diagnostics (51, 52), epidemiological surveillance (53–57), the definition of *S. aureus* outbreaks and the evolution of epidemic clones (46, 58–61), the identification of high-risk clones or genetic markers of antibiotic resistance, and the detection of genes encoding virulence factors (Fig. 1) (46, 57, 62, 63). Strains N315 and Mu50 were the first *S. aureus* genomes fully sequenced (64). Kuroda et al. used a shotgun random sequencing approach, in which genomic DNA is randomly and mechanically sheared into 1.0–2.2 kb fragments that are introduced into a plasmid vector (64). For this shotgun approach, a whole-genome plasmid library was generated in *Escherichia coli*, which in turn allowed for amplification and subsequent sequencing of genomic DNA fragments. In the last two decades, nucleotide sequencing has become more affordable and routine due to optimization of methods and, in particular, the introduction of high-throughput or next-generation sequencing (NGS) platforms, advances in computation, and increased instrument capacity. According to data from the National Human Genome Research Institute (65), the sequencing cost per megabase decreased from >$5,000 in 2001 to $0.006 in 2022. At present, ~2,400 complete and annotated *S. aureus* genomes and >108,000 annotated genomes at different assembly levels have been posted on the GenBank database at the National Center for Biotechnology Information (66). NGS encompasses multiple sequencing platforms that have their own specific protocols (e.g., Illumina NextSeq, HiSeq, SOLiD, 454 Roche). These NGS technologies, in general, rely on massively parallel sequencing of millions of short genomic DNA fragments (frequently 150–300 bp in length) that comprise single-stranded DNA libraries (67). Other NGS platforms used sequence capture arrays and whole-genome resequencing. Some of the first NGS studies with *S. aureus* elucidated the evolution of epidemic clones, such as the pandemic phage-type 80/81 clone (59), or informed on the transmission of outbreak strains within a healthcare facility (68).

WGS has greatly enhanced the utility of MLST, arguably the most widely used allele-based typing method. Introduction of a core genome MLST (cgMLST) that relies on a set of 1,861 targeted genes or its extension whole-genome MLST (wgMLST) that encompasses core and accessory genome has significantly increased the resolution and differentiation power of this typing method (62). Whether using cgMLST (MLST/wgMLST) or a method based on single-nucleotide polymorphisms (SNPs) relative to a reference strain (or set of strains), WGS facilitates highly reproducible and concordant epidemiological and phylogenomic analysis of clinical isolates (53, 69, 70). Additionally, WGS can deduce antibiotic resistance profiles of *S. aureus* clinical isolates with high correlation to standard antimicrobial susceptibility testing methods; >90% correlation in a large-scale study by Gordon et al. (71) and ~99% concordance rates in more recent studies, although the results are moderately antibiotic dependent (72–74).

The availability of complete genomic sequences for microorganisms such as *S. aureus* was a much-needed catalyst for the development of numerous high-throughput genetic platforms and methods, such as microarrays or RNA sequencing (RNA-seq). Although these NGS approaches contributed significantly to many areas of infectious diseases and microbial pathogenesis research, new approaches are in development or being used currently. Most notably, third-generation sequencing methods such as single-molecule real-time sequencing from Pacific Bioscience (PacBio) (75), Oxford Nanopore Technologies (ONT) sequencing (76), or Illumina Complete Long Read (ICLR) technology (77) produce reads up to hundreds of kilobases in size and are the next evolutionary step in genome sequencing approaches (78). These long-read sequencing (LRS) technologies are superior at resolving structural variants, such as large insertions, deletions, plasmids, and genome rearrangements, sequences that contain large numbers of repetitive elements, and DNA methylation sites. For example, Alves et al. used LRS to investigate the underlying genetic basis for phenotypic changes in *S. aureus* small colony variants that developed during intracellular survival in macrophages (79). This included assessment of DNA methylation in the macrophage-adapted small colony variants (79). Hybrid assembly approaches, which combine LRS and short-read sequencing, have been used to overcome problems associated with repetitive elements in *S. aureus*. For instance, Belikova et al. used hybrid genome assembly to identify heterogeneity among closely related USA300 isolates caused by gene copy number variants (80), and Panthee et al. used hybrid assembly to complete the RN4220 genome (81). In addition to the genome assembly utility, ONT has LRS systems designed for portability and rapid generation of sequence data, which have potential applications for bacterial surveillance, clinical epidemiology, diagnostics, and infection control (49, 82–84). Landman et al. evaluated this technology for surveillance of multidrug-resistant bacteria, including MRSA (49). Thus, there is potential for expanded use of LRS with *S. aureus* research and clinical applications, especially as read accuracy continues to improve (78, 83, 85, 86).

## MICROARRAYS

In brief, microarray technology refers to the high-throughput capability to perform thousands of assays in a serialized fashion in a small, confined vessel. A variety of microarray platforms have been utilized over the past three decades, including DNA and RNA microarrays (87), protein microarrays (88–92), and phenotype microarrays (93). For many years, microarrays were widely used to measure changes in global gene expression (transcriptome analyses) of host and/or microbial pathogens. DNA microarrays were utilized to evaluate the presence or absence of genes, insertions or deletions, and to detect SNPs (94). Both DNA and RNA microarrays are highly organized matrices of thousands of DNA products that rely on a probe-based hybridization approach to detect their target. Several microarray methods have been developed, but two techniques prevailed—spotted microarrays and oligonucleotide microarrays (95). These two techniques differ primarily in the size of DNA fragments used and the method used to attach these DNA fragments to the solid surface. For spotted microarrays, amplified and purified DNA fragments of interest (e.g., PCR product for open reading frames) are attached to a solid surface, such as coated glass slides, using a robotic array printer/spotter (87). Oligonucleotide microarrays or GeneChip probe arrays were developed by Affymetrix, Inc. (now Thermo Fisher) and are high-density arrays typically composed of ~20-mer oligonucleotides (96). The oligonucleotides are synthesized *in situ* onto hydroxyalkyl groups that are covalently attached to the surface of the microarray with high precision and spatial resolution. The process relies on a photolithographic mask that allows for photodeprotection of a specific area during the oligonucleotide synthesis (96). Both microarray platforms utilize a process whereby DNA or RNA material from test samples is labeled with fluorophores or biotin-dUTP and hybridized to DNA fragments on the array. For transcriptome analyses, the level of gene expression is determined based on the color and intensity of the fluorophores during detection (87).

Landmark studies by Fitzgerald et al. were among the first to use DNA-spotted microarrays for *S. aureus* research (97). This work provided conclusive evidence that MRSA strains had arisen multiple times by horizontal transfer of the *mec* element and shed light on the basis of the epidemic of toxic shock syndrome that occurred in the late 1970s (97). Subsequent studies used microarrays for MLST of *S. aureus* (98), comparative genome sequencing and phylogenetic analyses of the epidemic USA300 clone (99), and detecting virulence or antibiotic resistance determinants (100–103). Dunman et al. used custom-made Affymetrix GeneChips to identify transcripts regulated by *S. aureus agr* and *sarA* gene regulators, including identification of putative virulence molecules (102). These studies were among the first to measure changes in the *S. aureus* transcriptome and set a standard for the field moving forward (102). Microarray approaches have since contributed greatly to our understanding of *S. aureus* transcriptome dynamics during host-pathogen interactions, including interactions with host cells (103, 104), adaptations to blood and serum (105), lungs (106), or changes in gene expression in skin infections (107, 108).

## RNA SEQUENCING

Inasmuch as NGS methodologies are now routine and relatively inexpensive, microarray-based analyses of bacterial transcriptomes have been replaced largely by bulk RNA-seq. RNA-seq was developed by Nagalakshmi et al. (109) to study yeast gene expression, but it rapidly became a method of choice for transcriptome analyses. RNA-seq allows for unbiased profiling of global expression of genes in a test population at the time of collection and, importantly, does not rely on predetermined transcript- or species-specific probe sets. RNA-seq utilizes NGS to sequence all transcribed RNAs (transcriptome) simultaneously. Briefly, bacterial RNA is isolated from a sample, and genomic DNA and/or rRNA are removed. The enriched mRNA is fragmented and subsequently reverse-transcribed to generate a cDNA library for sequencing. The reads obtained from this process are aligned to a reference genome, and quantitative analysis of all expressed transcripts within a bacterial test population is performed based on the number of reads (read count) for each gene. RNA-seq has proven to be more accurate and sensitive than microarray-based approaches. The method does not have lower and upper saturation limits of detection that are intrinsic to microarrays, and therefore, RNA-seq permits detection of low-abundance transcripts and more subtle changes in expression levels (110, 111). RNA-seq avoids one of the major limitations of the microarray approach—namely, the need to separate RNA material from different species in mixed samples to avoid cross-hybridization. Indeed, RNA-seq permits studies of the *S. aureus* transcriptome in natural niches within polymicrobial communities, such as the anterior nares of persistently colonized people (112). The ability of this method to analyze RNA transcripts from different species led to the development of dual RNA-seq, which permits analysis of concurrent host and pathogen gene expression (113). Studies employing dual RNA-seq for *S. aureus* research reveal crosstalk between host and pathogen, as well as bacterial adaptation to the host response during infection (114, 115).

### Single-cell RNA sequencing

Microarray and RNA-seq approaches have contributed significantly to our understanding of the role of *S. aureus* gene expression *in vitro* and *in vivo* (102–108, 112, 114, 115). These methods provide the averaged gene expression of a test population but do not resolve differential responses within the population. Advances in sequencing methodologies led to the development of single-cell RNA sequencing (scRNA-seq), which enables assessment of the transcriptome at the single-cell level. Current bacterial scRNA-seq approaches can be grouped based on (i) a cell separation and barcoding step that typically involves a microplate (split-pool barcoding, see below) or a droplet-based fluidics approach, and (ii) RNA capture; a probe-based approach or whole transcriptome amplification with random primers.

scRNA-seq can determine heterogeneity of bacteria in a population and facilitates the investigation of the transcriptome of specific subpopulations of cells that otherwise could have been lost in bulk RNA analysis. In the context of *S. aureus* biology, such information could, for example, assist with the investigation of the molecular basis of hVISA. scRNA-seq of mammalian cells and tissue samples has progressed exponentially since its first implementation in 2009 (116). Despite this progress, bacterial scRNA-seq remains a challenge. A major issue with bacterial scRNA-seq is the amount and stability of mRNA within each cell. On average, mammalian cells contain 10–50 pg of total RNA, whereas bacterial cells contain ~100-fold less. Additionally, bacterial mRNA constitutes only a small portion of the total cellular RNA (<5%), and it is short-lived compared to that of mammalian cells. It is vital for bacteria such as *S. aureus* to adapt quickly to environmental stressors and varied growth conditions. This entails sensing and rapid production or degradation of certain molecules at the level of mRNA transcripts (117–120). With *in vitro* assay conditions, the amount of bacterial mRNA is typically growth phase dependent. For example, *S. aureus* mRNA turnover is high during the logarithmic phase of growth—approximately 90% of mRNA transcripts have a half-life of less than 5 min (121, 122). Eukaryotic scRNA-seq methods often enrich for mRNA transcripts by using the poly(A)-tail at the 3′ end of the transcript (116). This allows transcripts to be captured by poly-d(T) primers encoding a cell barcode and/or unique molecular identifier (UMI) sequence. Prokaryotic mRNA in general does not have the same poly(A)-tail. Bacterial polyadenylation of mRNA transcripts has low efficiency, and bacterial poly(A)-tails are significantly shorter compared to eukaryotic counterparts. Moreover, in eukaryotes, polyadenylation stabilizes mRNA transcripts and increases their potential for translation, whereas in bacteria, polyadenylation is seen rather as a degradation label (123, 124). Nonetheless, *in vitro* polyadenylation of bacterial mRNA could be used to enrich the mRNA pool, as described for some of the single-cell methods.

Bacterial scRNA-seq methodology depends in part (and to varying degrees) on permeabilization of the cell wall to accommodate enzymatic reactions such as labeling or reverse transcription of RNA. These processes must occur without compromising cell structure prematurely. Bacterial cell wall permeabilization frequently involves enzymatic digestion with lysozyme for gram-negative bacteria and lysostaphin or lysostaphin with lysozyme for gram-positive bacteria. Not all scRNA-seq approaches have been used successfully with *S. aureus* or other gram-positive bacteria. Recent bacterial scRNA-seq techniques that have been tested with *S. aureus* are shown in Table 1. There are several important steps associated with bacterial scRNA-seq. Given the short half-life of bacterial mRNA, cells are typically fixed by incubation with paraformaldehyde at the time of sample collection to immobilize and preserve RNA transcripts of interest. mRNA from each cell undergoes reverse transcription (RT) and is given a barcode and UMI. Following cell lysis, the library is prepared, and transcripts are amplified and sequenced.

**TABLE 1:** Current scRNA-seq methods^*a*^^,^^*b*^^,^^*c*^

Method	PETRI-seq (Prokaryotic expression profiling by tagging RNA in situ and sequencing)	microSPLiT (Microbial split- pool ligation transcriptomics)	RiboD-PETRI (rRNA-derived cDNA depletion-PETRI)	BaSSSh-seq (Bacterial scRNA-seq with split-pool barcoding, second-strand synthesis, and subtractive hybridization)	MATQ-seq (Multiple annealing and deoxycytidine tailing-based quantitative scRNA-seq)	BacDrop (Droplet- based genome-wide massively parallel bacterial scRNA-seq)	M3-seq (Single-cell massively- parallel, multiplexed, microbial sequencing)	ProBac-seq (Probe-based bacterial sequencing)	smRandom-seq (Single-microbe RNA-seq)
Cell input	~3 × 10^7^ for barcoding and ~ 10,000 for library prep	~1 × 10^6^ for barcoding and >25,000 for sequencing	30,000–60,000	15,000–25,000 cells library	Up to 96 cells per passage	~4 × 10^7^ for rRNA depletion	~ 100,000	~10,000	~10,000
Platform	96-well plate/split- pool barcoding	96-well plate/split- pool barcoding	96-well plate/split-pool barcoding	96-well plate/split- pool barcoding	Fluorescence-activated cell sorting	384-well plate first barcoding/ 10x Genomics	96-well plate barcoding/ 10x Genomics	mRNA specific probes/ 10x Genomics	Custom microfluidics platform
Cell wall permeabilization	Lysozyme/ lysostaphin	0.04% Tween-20/lysozyme	Lysozyme/lysostaphin	Lysostaphin	Lysozyme	0.04% Tween-20/ lysozyme	Lysozyme	MAAM/lysozyme	0.04% Tween-20/ lysozyme
Doublet or collision rate	1.5%	Up to 5%	1.16%–3.35––%	–	0%	6.6% per two million cells	Up to 32% collision rate	2.8%	1.6%
Double-stranded cDNA generation	Second-strand synthesis	Template switching; Maxima H	Template switching; Maxima H	Second-strand synthesis	G-enriched MALBAC primers (poly(C)-tailing	Second-strand synthesis; dA- tailing	Second-strand synthesis	Probes with poly(A)-tail; 10x Genomics chemistry	Second-strand synthesis; dA-tailing
rRNA depletion	n/a	n/a; poly(A)-tailing	RiboD (RNase H and probe base subtractive hybridization)	Dual-strand probe base subtractive hybridization	Depletion of abundant sequences by hybridization (DASH; Cas9 mediated)	RNase H	rRNA specific probes followed by RNase H digestion	n/a; mRNA specific probes	DASH (Cas9 mediated)
Sensitivity for G− (per cell)	20–200 mRNA transcript for *E.c*.	*E.c.*: median of 235 mRNA UMIs (138 genes)	*E.c.*: median of 102 UMIs *C.c.*: median of 182 UMIs	–	*S.e*.: average of 307 genes *P.a*.: average of 102 genes	*E.c*.: average of 90 genes *K.p*.: average of 90 genes	*E.c*.: up to 211 UMIs (151 genes)	*E.c*.: median of 263 transcripts (165 genes)	*E.c*.: median of 428 UMIs (225 genes) *A.b*.: 307 UMIs (204 genes) *K.p*.: 610 UMIs (321 genes) *P.a*.: 324 UMIs (245 genes)
Sensitivity for G+ other than *S.a.* (per cell)	–	*B.s*.: median of 397 UMIs (230 genes)	–	–	–	Tested in *E.f*.	*B.s*.: up to 984 UMIs (371 genes)	*B.s*.: median of 325 mRNA transcripts (241 genes) *C.p*.: median of 153–215 mRNA transcripts	*B.s*.: median of 325 mRNA transcripts (1,249 genes)
Sensitivity for *S.a.* (per cell)	Median of 43 mRNA transcripts	–	Median of 142 UMIs	Average of 34 for biofilm and 60 mRNA reads for planktonic culture	–	–	Tested but not optimized	–	Median of 393 UMIs (206 genes)
Reference	128	129	130	131	125-127	137	136	138, 139	140

^*a*^ Gram-negative (G−) bacterial species: *A.b.*,* Acinetobacter baumannii*; *C.c.*,* Caulobacter crescentus*; *E.c.*,* E. coli*; *K.p.*,* Klebsiella pneumoniae*; *P.a.*,* Pseudomonas aeruginosa*; *S.e.*,* Salmonella enterica. *

^*b*^ Gram-positive (G+) bacterial species: *B.s.*,* Bacillus subtilis*; *C.p.*,* Clostridium perfringens*; *E.f.*,* Enterococcus faecium*; *S.a.*,* S. aureus. *

^*c*^ n/a, not applicable; –, information not found.

One of the early scRNA-seq protocols utilizes multiple annealing and deoxycytidine (dC) tailing-based quantitative scRNA-seq (MATQ-seq) (125). MATQ-seq uses fluorescence-activated cell sorting to achieve a single-cell suspension in a multi-well plate format and does not rely on barcoding strategy. To increase the efficiency of reverse transcription and second-strand synthesis, MATQ-seq employs random primers and low-temperature multiple annealing cycles without intermediate melting steps; annealing temperature is increased during each consecutive cycle (125). This method was labor intensive, and there was relatively low throughput in terms of cell numbers. More recently, an improved bacterial MATQ-seq protocol added the automation of several steps as well as a Cas9-based rRNA removal protocol (126, 127). This improved methodology increased transcript recovery and reduced workflow and hands-on time (127).

### Split-pool barcoding

Split-pool barcoding (also known as combinatorial indexing) approaches use fixed and permeabilized cells as individual compartments for barcoding steps and other enzymatic reactions. The cell suspension is typically split into wells of multiwell plates for the RT step with a random well-specific barcoded primer. After initial RT, cells are pooled and redistributed into a new multiwell plate for an additional round of barcoding by ligation. If required, this step can be repeated one more time, resulting in a unique three-barcode combination for the cDNA from each cell. A split-pool barcoding method—among the first to be employed—known as PETRI-seq (prokaryotic expression profiling by tagging RNA *in situ* and sequencing) was tested with the USA300 *S. aureus* strain (128). This is a high-throughput method that allowed for barcoding of 10^7^ cells per reaction, and ~10^5^ cells were used for library preparation and sequencing. Additionally, second-strand synthesis was used instead of a template switch method to increase double-stranded cDNA yield. Overall, without any rRNA depletion, about 15% and 25% of sense UMIs mapped to mRNA for exponentially grown *E. coli* and *S. aureus*, respectively. This method resulted in a median of 43 mRNA transcripts per *S. aureus* cell (128). The authors demonstrated that PETRI-seq can detect heterogeneity in the transcriptome profile of bacterial cells in the culture and had sufficient resolution to detect subpopulations equivalent to 0.04% of total *S. aureus* cells that were undergoing prophage induction (128).

Microbial split-pool ligation transcriptomics (microSPLiT) is a split-pool barcoding method developed around the same time as PETRI-seq (129). MicroSPLiT adds an *in situ* polyadenylation step prior to split-pool barcoding. This process uses an anchored poly(dT) barcoded RT primer in addition to random hexamer during the first round of barcoding and ultimately results in a 2.5-fold mRNA pool enrichment. This approach resulted in 235 and 397 mRNAs per cell for *E. coli* and *Bacillus subtilis*, which are 3.7% and 9.5% of total cellular RNA (129). In general, mRNA UMIs resulting from these split-pool barcoding methods represent a relatively low percentage of total RNA transcripts recovered, compared to those recovered by methods employing rRNA removal techniques (130).

Recently, Yan et al. published a modified version of PETRI-seq termed RiboD-PETRI, which uses biotin-labeled probe primers that target rRNA-derived cDNA (130). During the library construction step, total cDNA is treated with RNase H followed by hybridization with probe primers and biotin-labeled universal primers. This allows for streptavidin-based purification of probes and bound rRNA-derived cDNA depletion (RiboD) to increase mRNA recovery. The authors used a template switching method for the cDNA library construction step vs the second-strand approach originally used in PETRI-seq (128, 130). Yan et al. showed that the RiboD step enriched mRNA transcripts significantly, from 10% to 92% of total transcripts for the stationary phase *S. aureus* and resulted in a median of 154 UMIs per cell. This method proved capable of detecting different subpopulations of *S. aureus* during the stationary phase of growth in liquid media. Additionally, RiboD-PETRI highlighted the heterogeneity of *E. coli* biofilms and identified a potential marker for persister cell populations (130).

Bacteria in a biofilm are potentially less transcriptionally active than those in planktonic culture, and the use of scRNA-seq for such studies has only recently become technically feasible. Korshoj and Kielian developed a novel scRNA-seq method, named BaSSSh-seq (bacterial single-cell RNA sequencing with split-pool barcoding, second-strand synthesis, and subtractive hybridization), that has been adapted specifically for studying *S. aureus* in biofilms (131). Recovery of mRNA transcripts with BaSSSh-seq is increased by the use of second-strand synthesis for cDNA library preparation and dual-strand subtractive biotin-probe hybridization to remove rRNA. BaSSSh-seq revealed greater heterogeneity among bacteria in a biofilm compared to planktonic culture and identified changes in gene expression unique to specific cell clusters (131). Furthermore, these researchers discovered that *S. aureus* in biofilms alters transcriptional responses based on the type of immune cells that are encountered. For example, co-incubation of *S. aureus* with bone-marrow-derived mouse macrophages elicited changes in transcripts such as *perR* (peroxide resistance) and *nos* (encoding nitric oxide synthase) that encode proteins involved in the response to oxidative stress and/or were reported previously to promote *S. aureus* survival in the context of host phagocytes or during infection *in vivo* (131–134). Co-incubation with murine neutrophils also caused changes in *S. aureus* transcripts involved in oxidative stress but additionally triggered changes in genes encoding proteins that contribute to cell wall metabolism and virulence (131). BaSSSh-seq is an example of an important technological advance that generated an enhanced view of *S. aureus* biology.

### Droplet-based microfluidics

Droplet-based microfluidics methods are founded on the encapsulation of a single cell in a droplet, which enables unique barcoding of cell RNA transcripts. These droplets become unique compartments for enzymatic reactions. Several methods take advantage of commercially available microfluidics platforms developed by 10x Genomics (135). Such platforms are widely used for eukaryotic scRNA-seq studies. For example, M3-seq (single-cell massively-parallel, multiplexed, microbial sequencing) (136) and BacDrop (droplet-based genome-wide massively parallel bacterial scRNA-seq technology) (137) combine a multi-well plate barcoding approach and the 10x Genomics platform. Both techniques utilize two rounds of cell indexing. The first round uses split-pool barcoding by *in situ* reverse transcription with random primer, which also tags transcripts with a UMI. Cells are then combined and loaded into a 10x Genomics microfluidics platform for a second round of barcoding. Although M3-seq and BacDrop each use RNase H to remove rRNA, a step that increases the relative amount of non-rRNA transcripts to >80% of total RNA, the timing of this step in each workflow is vastly different. For BacDrop, rRNA and subsequent DNA removal happen *in situ* immediately after cell fixation and permeabilization and prior to any RNA manipulation (137). By comparison, rRNA removal with M3-seq is performed after amplification of cDNA libraries and transcription to single-stranded RNA. Specific DNA probes are used to hybridize rRNA, which is then followed by removal with RNase H. These rRNA-depleted libraries are then reverse transcribed to cDNA for sequencing (136). The M3-seq protocol was tested with *S. aureus*, but the UMI capture rate for *S. aureus* was suboptimal compared to the other two bacterial species tested (*E. coli* and *Bacillus subtilis*) (136). More work is needed to optimize M3-seq for use with *S. aureus*.

A method known as probe-based bacterial sequencing (ProBac-seq) combines custom single-stranded DNA probes that target several 50 bp regions within each open reading frame of the mRNA transcripts of fixed and permeabilized cells with Chromium Controller from 10x Genomics (138, 139). The probes have a 5′ end PCR handle for the generation of the library and UMIs and a 3′ end poly(A)-tail that allows compatibility with the 10x Genomics platform. The use of sequence-specific probes that hybridize to mRNA eliminates the need for rRNA removal.

A single-microbe RNA-seq method termed smRandom-seq is a droplet-based high-throughput single-microbe RNA-seq assay that deviates from the use of the 10x Genomics microfluidics platform and instead utilizes a custom system (140). Although the method was originally developed for use with *E. coli*, it has been validated for use with gram-positive and gram-negative bacteria, including *S. aureus*. Prior to encapsulation, fixed and permeabilized bacteria undergo *in situ* reverse transcription with random primers equipped with a GAT 3-letter PCR handle to capture total RNA. This process is followed by (dA)-tailing at the 3′ end for subsequent second-strand cDNA synthesis. Barcoding and a UMI tagging step were performed upon bacterial encapsulation into droplets with poly(dT) beads. Using a custom microfluidics platform permitted adjustments in the droplet size and consequently more efficient barcoding of low-content RNAs. To remove rRNA-derived cDNA, these researchers used a previously described Cas9-based depletion of abundant sequences by hybridization (126). Incorporation of this depletion step increased mRNA abundance from 16% to 63% of total RNA for *E. coli* or from 7% to 41% for *S. aureus* (140).

### Caveats

Each of the scRNA-seq methods described here has advantages and disadvantages. Split-pool barcoding methods can process considerable numbers of cells simultaneously and do not require specialized equipment. However, these methods rely on the integrity of fixed and permeabilized cells throughout multiple steps, which represents a challenge. Split-pool barcoding scRNA-seq methods have relatively low cell recovery (30%–50%), which potentially can introduce unintentional bias to the recovered pool of cells and sequenced transcripts. Cell recovery may also vary based on bacterial species and subsequent cell wall structure. On the other hand, methods based on a microfluidics approach are not limited by the constraints of a multiwell plate format or the stability of bacterial cell walls during enzymatic reactions and can process larger numbers of cells. Nonetheless, these latter methods impose additional costs associated with the microfluidics platform. There are also varied strategies for barcoding, use of random primers or specific probes for RT steps, second-strand cDNA synthesis vs a template switching technique, or rRNA depletion and its implementation within a workflow. The selection of the optimal method will depend on the bacterial species, experimental design, and accessibility of reagents or technology.

## CONCLUSIONS AND FUTURE DIRECTIONS

Bacterial genomics approaches have advanced tremendously over the last three decades. These methodologies have improved our understanding of *S. aureus* biology, evolution, and pathogenesis, and they have enhanced our ability to track *S. aureus* outbreaks and implement new infection control measures. Although significant progress has been made, our understanding of heterogeneity within populations of *S. aureus*, whether during growth *in vitro* or during infection *in vivo*, remains largely undetermined. We anticipate that single-cell sequencing approaches and associated technological advancements will facilitate an enhanced understanding of mechanisms underlying the expansion and establishment of different bacterial subpopulations under specific environmental conditions. In-depth understanding of bacterial population dynamics at the single-cell level, such as that for bacterial persister cells (141–143) (which may include hVISA/VISA), can potentially guide more successful use of antibiotics. Moreover, single-cell sequencing has the potential to highlight the events that lead to specific host adaptations by *S. aureus*, such as host range adaptability, clonal expansion of isolates during persistent infections (144), and the ability of *S. aureus* subpopulations to evade killing by host immune cells (79). The application of spatially resolved transcriptomics technologies with subcellular resolution presents an opportunity for unprecedented visualization of bacteria-host interactions (145, 146). Methods such as bacterial-MERFISH (147), seqFISH by SpatialGenomics or the Xenium *In Situ* platform (10x Genomics) (148), which allows for an add-on panel of custom probes that can target microbial RNA, provide a solid foundation for further assay development. These new technologies have to first be adapted for use with *S. aureus*, but mapping intercellular interactions—whether within specific organs during infection, or among microbial communities—has the potential to highlight nuances of functional heterogeneity of bacterial populations and modes of their adaptability to the environment. Collectively, these technologies have the potential to address important unresolved questions in many areas of *S. aureus* research. A few selected examples are provided below.

*Antibiotic resistance*

- What genes are essential for the development of *S. aureus* persister cells during treatment with antibiotics?

*Asymptomatic colonization*

- How does *S. aureus* alter gene expression at the subpopulation level to compete with other bacterial species within a microbiome?
- What molecular processes at the single-cell level are important for a switch from an asymptomatic colonizer to an invasive pathogen?

*Host-pathogen interactions*

- What are the host and bacterial factors that contribute to the establishment of infection in specific organs/host target sites? Such information can assist in the identification of optimal therapeutic targets.
- How does *S. aureus* establish a new host niche or colonize a novel host?
- What are the host molecular determinants (other than those associated with known immune deficiencies and susceptibilities) that determine the success or failure of *S. aureus* infection?
- What is the molecular basis of the differential phagocytic capacity of *S. aureus* among neutrophils from the same individual? This question is largely directed at the role of neutrophil heterogeneity.
- What is the mechanism of human neutrophil lysis following phagocytosis of *S. aureus*? Which *S. aureus* molecules (if any) contribute to this process directly?

We are still in a relatively early stage of development for bacterial single-cell sequencing technologies, and improvements in current methods are needed. Improvements to sample preparation steps, as well as advancement of the analytical components, bring us invariably closer to providing answers about *S. aureus* adaptability and antibiotic resistance stemming from population heterogeneity.
